# Exploring OCD severity in treatment-seeking veterans: a cross-sectional comparison between post-traumatic stress disorder (PTSD) and complex-PTSD (C-PTSD)

**DOI:** 10.1186/s40359-025-02446-0

**Published:** 2025-04-23

**Authors:** Phoebe Howlett, Theadora Lilian Rose Sudera, Natasha Biscoe, Jo Billings, Dominic Murphy

**Affiliations:** 1 Combat Stress, Tyrwhitt House, Oaklawn Rd, Leatherhead, KT22 0BX UK; 2 Division of Psychiatry, University College London, Gower Street, London, WC1E 6BT UK; 3 King’s Centre for Military Mental Health, King’s College London, Cutcombe, London, SE5 9PR UK

**Keywords:** Military veterans, Obsessive compulsive disorder (OCD), Complex post-traumatic stress disorder (C-PTSD), Post-traumatic stress disorder (PTSD), Disturbances of self-organisation (DSO)

## Abstract

The recent International Classifications of Diseases-11 (ICD-11) distinction of complex- post-traumatic stress disorder (C-PTSD) from post-traumatic stress disorder (PTSD), has highlighted a research gap in exploring how C-PTSD may relate to obsessive-compulsive disorder (OCD) differently than PTSD. Mental health disorders and comorbidities appear to be greater in military veterans compared to the general population. Thus, this study aimed to explore potential differences in OCD severity between probable PTSD and probable C-PTSD in a national clinical sample of UK military veterans. Data from 428 veterans were analysed using a previously collected dataset. The survey assessed sociodemographic characteristics, military experiences, physical and mental health, and well-being. Results indicated significant differences in OCD severity between probable PTSD and probable C-PTSD. OCD severity significantly increased as C-PTSD symptom severity increased for veterans with probable C-PTSD. Though no significant association was identified between OCD severity and PTSD scores within the probable PTSD group, this finding should be interpreted with caution, as the small sample may have limited statistical power. Greater C-PTSD severity significantly predicted greater OCD severity, but PTSD scores did not. Disturbances of self-organisations (DSO) symptoms within C-PTSD were more strongly associated to OCD severity compared to PTSD symptoms, indicating a seemingly complex interplay between C-PTSD’s cluster of symptoms and OCD severity. Future research should focus on replication involving larger veteran samples and the general population, incorporating clinician-administered assessments alongside self-report measures to enhance diagnostic accuracy.

## Introduction

Obsessive compulsive disorder (OCD) is a debilitating mental health condition effecting approximately 1–3% of the global population [1]. It is characterised by obsessive thoughts and/or compulsive behaviours [2]. Obsessive thoughts can be intrusive and arise involuntarily, often centring around distressing impulses, images, or ideas [2]. To alleviate these obsessions, compulsive behaviours are performed and are either not related in a realistic manner to the feared event or are clearly excessive [2]. Importantly, research indicates OCD to be likely underrecognized and inadequately treated within veterans [3], demonstrating the importance of research in this domain.

OCD is often experienced alongside comorbid psychiatric conditions, whereby a recent systematic review estimated a comorbidity rate of 69% in individuals with OCD [4]. With non-response to treatment often involving the presence of comorbid conditions [5], assessing OCD comorbidities and their implication in the management of OCD is paramount. Specifically, the prevalence rate of OCD is considerably higher among those with post-traumatic stress disorder (PTSD) [6]. Though the estimates of comorbidity of OCD and PTSD vary, estimates range from about 19% [7] to 31% [8] which is much larger than 1–3% in the general population [1]. Investigating psychiatric conditions that potentially influence OCD severity is vital for informing preventative interventions and treatment options.

PTSD is a complex mental health condition that can occur after traumatic life events and is characterised by intrusive re-experiencing, avoidance, and hyperarousal [2]. The increased co-occurrence of PTSD and OCD may be due to potential parallels between the symptoms, whereby both include repeated intrusive thoughts and avoidant behaviour that interfere with functioning and are driven by the need to avoid any cue, object, or place that might cause distress [2]. Specifically, cognitive models of OCD highlight the misinterpretation of intrusive thoughts as significant threats which potentially lead to compulsive behaviours aimed at neutralizing such thoughts [9, 10]. Similarly, individuals with PTSD often exhibit a heightened sensitivity to distressing thoughts and memories, which may lead to a similar pattern of avoidance or compulsive behaviours [2]. This highlights the potential shared vulnerability to anxiety and maladaptive coping strategies in both OCD and PTSD [11]. A meta-analysis indicated how exposure to past trauma is associated with a higher severity of obsessive-compulsive symptoms, particularly compulsions [12]. Some research indicates this relationship may occur due to OCD symptoms acting as safety behaviours against the challenging symptoms of PTSD [13]. The cognitive theory of OCD also suggests that obsessions are more likely to occur when an individual is exposed to stressful situations and that external cues often trigger obsessional thoughts [14], indicating a potential link between OCD and trauma. Considering the increased co-occurrence of PTSD and OCD, understanding the complexities of comorbidity is essential for tailored treatment options.

The sibling diagnosis to PTSD, complex post-traumatic stress disorder (C-PTSD) was recognised as a unique mental health condition in 2018 in the International Classification of Diseases (ICD-11) [15]. According to the ICD-11, PTSD and C-PTSD are two different diagnoses and thus C-PTSD is not simply an intensified form of PTSD [15]. C-PTSD includes the same symptoms as PTSD as well as an additional cluster of symptoms related to disturbances of self-organisation (DSO) [16]. The DSO symptoms in C-PTSD include affective dysregulation, negative self-concept, and disturbances in relationships [17]. While C-PTSD can develop from a single traumatic event, it is commonly associated with exposure to repetitive or multiple traumatic events, whereby escape is difficult or impossible [18]. Latent profile analysis suggests C-PTSD as having a distinct symptom profile compared to PTSD, with greater functional impairment [19, 20].

A recent cross-sectional study found a high prevalence of probable PTSD (69%) among treatment-seeking veterans, with the majority of participants showing probable C-PTSD (63%) compared to probable PTSD (6%), based on self-reported assessments [21]. Additionally, a recent systematic review of serving military and veteran populations reported probable C-PTSD prevalence rates ranging from 5 to 80.63%, compared to probable PTSD prevalence rates spanning from 3.8 to 42.3% [22]. This systematic review included a total of 16 studies, whereby 13 of the 16 demonstrated a higher prevalence of probable C-PTSD compared to probable PTSD. Notably, only one of the studies indicated a higher prevalence of probable PTSD (36.7%), compared to probable C-PTSD (16.4%) with this study including active serving Filipino soldiers [23]. This study was one of the two studies in the review that incorporated active soldiers with all the remaining including veterans. The cross-sectional study [21] and majority of the studies in the systematic review (i.e., 12 out of 16) used the International Trauma Questionnaire (ITQ), which is an established and reliable self-report measure [17]. As the ITQ was not designed to make a diagnosis without a clinical interview [24], it is important to note that the prevalence rates are not clinician informed. As such, these diagnoses should be considered probable rather than definitive. Interestingly, the studies in the systematic review which did not use the ITQ indicated prevalence rates of PTSD and C-PTSD which were much closer than other studies [25, 26, 27]. However, these studies also incorporated self-report measures. Given the emerging nature of C-PTSD research, findings across studies suggest that C-PTSD may be as prevalent, if not more so, than PTSD in veteran populations. Understanding the co-occurrence of C-PTSD and disorders such as OCD is particularly important in veterans, who exhibit higher rates of these conditions [23, 30]. Exploring these relationships could improve veterans’ quality of life post-service and provide broader insights into the interrelationship between C-PTSD, PTSD, and OCD.

Although this field of research is still emerging, largely due to the relatively recent updates to the C-PTSD diagnosis, results offer insight into how C-PTSD may be more prevalent than PTSD within the veteran population. Exploring the co-occurring relationship between C-PTSD and OCD is especially important in the veteran population due to their reportedly high rates of developing C-PTSD, PTSD and OCD [21, 28]. Understanding these interactions could contribute to veterans’ quality of life after service, whilst providing valuable insights into the broader understanding of the interrelationship between C-PTSD, PTSD, and OCD.

To our knowledge, no research has yet explored whether the DSO symptoms that distinguish C-PTSD from PTSD may play an important role in influencing OCD severity. However, prior literature has acknowledged how OCD may interfere with one’s self-concept and functional impairment related to interpersonal relationships [29, 30, 31], both of which are related to DSO symptoms. Research found OCD symptoms and maladaptive beliefs are exacerbated in individuals with a sensitive self-concept particularly when they perceive incompetence in domains of morality, job, scholastic and social acceptability [32]. As proposed in the cognitive theory of obsession, interpretating intrusive thoughts as personally significant is a key mechanism in this relationship, as individuals with sensitive self-concept may experience increased anxiety and preoccupation [9, 10]. As such, the DSO symptoms and sensitive self-concept in C-PTSD may lead to vulnerabilities to OCD symptoms such as cognitive rigidity, perfectionism, avoidance, increased sense of responsibility and rumination [30]. Similarly, functional impairment in interpersonal relationships of one’s work, family and social life has been associated with greater OCD severity [33]. Results inferred impairment in social relationships, including social support and disability within social environments, relates to more severe OCD. Research shows that individuals who seek interpersonal reassurance tend to experience more severe OCD compulsions [34], potentially reinforcing their symptoms of fostering dependency within relationships. Though these findings do not establish a direct link to DSO symptoms, they illustrate how OCD may interact with one’s self-concept and social functioning, both characteristics found within DSO. Thus, making it plausible to suggest DSO symptoms may be associated with OCD severity.

Military service not only affects veterans’ trauma-related psychiatric conditions but also appears to have a detrimental impact on social relationships with veterans experiencing an increased risk of loneliness and interpersonal difficulties [35, 36]. Additionally, evidence suggests veterans with mental health conditions, including PTSD and OCD have impaired social functioning [37, 38]. This literature demonstrates veterans’ vulnerability to social impairment. Concerning C-PTSD, this is significant because the distinguishing DSO symptoms involve disturbances in relationships, making it plausible to suggest an interplay between the high rates of C-PTSD and impairments in social functioning. In relation to OCD, this is relevant due to research indicating an association between OCD severity and significantly lower social functioning [33]. Understanding whether C-PTSD is associated with more severe OCD symptoms than PTSD may have important clinical and practical implications, particularly in highlighting the need for tailored interventions to improve treatment outcomes for individuals with comorbid conditions.

### Aims of this research

The primary aim of this study was to explore the potential difference in OCD severity between probable PTSD and probable C-PTSD within treatment seeking UK military veterans. Hypothesis one predicts military veterans with probable C-PTSD will have greater OCD severity compared to veterans with probable PTSD and No PTSD.

If a significant relationship from hypothesis one was found, then secondary analysis would explore whether OCD severity was associated with both PTSD and DSO cluster of symptoms within C-PTSD. Hypothesis two posited that greater PTSD and DSO symptoms within C-PTSD would be associated with greater OCD severity.

## Methods

### Setting, participants, and procedures

This study used a cross-sectional study design, which re-uses data that has previously been collected by Combat Stress [21], a UK mental health charity for military veterans. This original survey has been used in two other studies, where one explored the association between each C-PTSD symptom cluster and executive functioning [39], and one explored the prevalence of probable insomnia disorder and associated outcomes [40]. However, the current study aims to explore the association between OCD severity and probable PTSD and C-PTSD, providing a unique perspective that sets it apart from previous publications. This study conducted subsidiary analysis based on data extracted from the Patient Needs Survey; a survey constructed by Combat Stress to assess the health and well-being of treatment seeking veterans [21].

While the full population and methodology is provided in the previous paper [21], in brief, participants were contacted if they had; (1) received support from Combat Stress over a one-year period, (2) given informed consent to be contacted for research purposes, and (3) provided an email address to be contacted. Participants were treatment-seeking veterans, which was defined as having at least one treatment session with Combat Stress. Veterans were defined as having served at least one day of paid employment in the U.K. Armed Forces. A randomised sub-sample of 50% (*N* = 1,147) of the total veteran population who had received support from Combat Stress and expressed a willingness to participate in research, were invited to take part. This sample was selected to ensure an appropriate sample size for the study, while avoiding the risk of saturation of the entire contactable population with requests to take part in research. Of the sample of 1,147 veterans, 158 veterans were removed due to an invalid email address. Consequently, 989 veterans contacted by the research department. Data was collected via an online survey between August and September 2020. Five email invitations were sent to applicable individuals across a six-week period. Subsequently, all non-responders were sent the survey via post. 428 individuals responded to the Patient Needs Survey, out of the 989 invited to participate, totalling a response rate of 43.8%.

Data available for both responders and non-responders included age, sex, and service branch (Royal Navy, Army, Royal Air Force). Statistical tests (i.e., T-tests) were conducted to assess potential demographic differences between the responders and non-responders [21]. Results indicated that responders were found to be significantly (*t* = 4.78, *p* <.05, 95% CI: 3.01 to 7.24) older than non-responders, with a mean age of 50.5 years for responders and 44.3 years for non-responders. No significant differences were found between the groups in terms of sex or military branch. Notably, both responders and non-responders were more likely to be male (96.6% non-responders; 97.4% responders) and more likely to have served in the Army (85.9% non-responders, 82.5% responders) compared to other military branches. To eliminate potential overlap between previous studies that have conducted subsidiary analyses on the original survey conducted by Combat Stress [21], only participants that responded to the relevant sections central to the current study were included in the analyses. As such, of the 428 responders, 315 responded to the relevant sections of the survey.

### Ethical approval

The current project was an audit to understand the clinical needs of the veterans engaged with Combat Stress. Approval for this project was granted by Combat Stress Research Committee (ref.pn2020). The study was conducted in alignment with the Declaration of Helsinki for medical research with human participants. All participants had previously provided informed consent for their data to be used for audit purposes.

### Measures

#### The patient needs Survey

The Patient Needs Survey is a 32-question survey, devised by Combat Stress, consisting of eight sections. Sections 1 and 2 focused on collecting data on socio-demographic characteristics and military experiences. The subsequent sections focused on specific health and well-being factors measured using standard psychometric scales. The sections were: (1) About You, (2) Your Military History, (3) Questions About Your Social Network, (4) Questions About Your Gambling and Drinking Habits, (5) Questions About Your Health, (6) Questions About Obsessions and Compulsions, (7) Questions About Symptoms Related to a Stressful Event, and (8) Questions About Your Life Growing Up.

For this study, data was extracted from Sections 1, 6 and 7 as they focused on the key factors relevant to this study (i.e., sociodemographic characteristics, OCD symptoms, and PTSD symptoms). Sections 2, 3, 4, 5, and 8 were not deemed relevant to the current scope.

- Section 1 collected data on sociodemographic characteristics including, age, sex, ethnicity, relationship status, and employment status. These questions were constructed by Combat Stress.
- Section 6 explored OCD using The Yale-Brown Obsessive Compulsive Scale (Y-BOCS) [41]. The Cronbach’s alpha for the Y-BOCS was 0.91. Scores ranged from 0 to 40, with higher scores indicating greater OCD severity.
- Section 7 measured participants experience of trauma using The International Trauma Questionnaire (ITQ) [17]. The Cronbach’s alpha for the ITQ was 0.92. Section A explored PTSD symptoms within the last month, the symptom clusters included: re-experiencing, avoidance, and sense of current threat. Scores ranged from 0 to 36, with higher scores denoting greater PTSD symptoms. To classify a self-diagnosis of PTSD, individuals must obtain one of the two symptoms from each symptom cluster (i.e., re-experiencing, avoidance and sense of current threat), with at least one pointer of functional impairment associated with these symptoms, classified by a score of ≥2 [17]. Section B explored disturbances of self-organisation (DSO) cluster of symptoms, these included affective dysregulation, negative self-concept, and disturbances in relationships, scores ranged from 0 to 36. For a self-diagnosis of C-PTSD, individuals need to fulfil the PTSD criteria along with one of the two symptoms from each DSO cluster of symptoms, with at least one score indicating functional impairment classified by a score of ≥2 [17]. As such, an individual can receive a diagnosis of PTSD or C-PTSD, but not both.

#### The current study

Using diagnostic criteria based on ITQ scores described above [17], participants were categorised into the following diagnostic groups; (1) probable PTSD, (2) probable C-PTSD, and (3) no PTSD. ITQ scores were also used as continuous measures. ITQ-PTSD scores were included from section A, ITQ-C-PTSD included scores from section A and B, and ITQ-DSO included scores only from section B.

#### Analysis

Using descriptive statistics, the first part of the analysis described participant demographic characteristics and health and well-being variables across diagnostic groups. To assess whether there were significant differences in demographic characteristics across diagnostic groups, an ANOVA and chi-squared tests were performed.

The primary analysis assessed whether Y-BOCS scores differed between the three diagnostic groups, probable PTSD, probable C-PTSD, and No PTSD. An analysis of variance (ANOVA) was conducted using diagnostic groups (ITQ-PTSD, ITQ-C-PTSD, and No PTSD) as the predictors of Y-BOCS scores (the outcome). Next, two simple linear regression models assessed (1) whether Y-BOCS scores were predicted by ITQ-PTSD scores in participants with probable PTSD, and (2) whether Y-BOCS scores were predicted by ITQ-C-PTSD scores in participants with probable C-PTSD. In all these regressions, the ITQ scores (ITQ-PTSD and ITQ-C-PTSD) were the predictor variables and the Y-BOCS scores were the outcome variables.

Hypothesis 2 explored whether Y-BOCS scores for those with probable C-PTSD were associated with the PTSD cluster of symptoms (ITQ-PTSD) or DSO cluster of symptoms (ITQ-DSO); the data was split to separate ITQ scores for only those meeting probable C-PTSD diagnosis. Y-BOCS scores being a continuous variable permitted two simple linear regression models; one with ITQ-PTSD scores predicting Y-BOCS scores, and the other with ITQ-DSO scores predicting Y-BOCS scores. Following this, a multiple linear regression examined whether both ITQ-PTSD and ITQ-DSO were associated with Y-BOCS scores when included in the same model, or whether one acted as a proxy. Both simple and multiple linear regressions were conducted to explore the unique and combined relationship between ITQ-PTSD, ITQ-DSO, and Y-BOCS scores. Participants who had missing data on any of the relevant measures (measures in Sect. 1, Sect. 6, and Sect. 7) were excluded from the analysis. All analysis was conducted using R Studio (Version 4.2.2).

## Results

### Sample characteristics

428 individuals responded to the Patient Needs Survey, out of the 989 contacted. Of this 428, 315 responded to all of the questions in Sections 6 and 7 of the survey and were included in this study. Participants were predominantly male (97%), and of white race (94%). 43% of participants were not working, 40% were working, and 17% were retired. 66% of participants were in a relationship. Out of the 315 participants, 21 met diagnostic criteria for probable PTSD, 197 met diagnostic criteria for probable C-PTSD, and 97 met no probable diagnosis for PTSD. See Table 1  for further details of participant characteristics. Notably, no significant differences were found (*p* >.05) between the diagnostic groups in key demographic variables, including, age, sex, ethnicity, employment status, and relationship status.

**Table 1:** Descriptive Statistics of Sample Demographic Characteristics and Mental Health Scale measurements based on diagnosis

		All Participants (*N* = 315)			Probable PTSD (*N* = 21)			Probable C-PTSD (*N* = 197)			No PTSD (*N* = 97)		
Variable	Scale	*N* (%)	M	SD	*N* (%)	M	SD	*N* (%)	M	SD	*N* (%)	M	SD
Age			50.77	10.69		49.73	12.88		50.57	10.65		51.41	10.35
Sex													
	Female	9 (2.86%)			1 (4.76%)			6 (3.05%)			2 (2.06%)		
	Male	306 (97.14%)			20 (95.24%)			191 (96.95%)			95 (97.94%)		
Ethnicity													
	White	294 (93.93%)			19 (90.48%)			180 (92.31%)			95 (97.94%)		
	Minority	19 (6.07%)			2 (9.52%)			15 (7.69%)			2 (2.06%)		
Employment status													
	Working	126 (40.00%)			7 (33.33%)			69 (35.03%)			50 (51.55%)		
	Retired	55 (17.68%)			3 (14.29%)			37 (19.07%)			15 (15.63%)		
	No work	134 (43.09%)			11 (52.38%)			91 (46.91%)			32 (33.33%)		
Relationship													
	Yes	207 (66.35%)			17 (80.95%)			123 (62.43%)			67 (69.07%)		
	No	108 (34.29%)			4 (19.05%)			74 (37.56%)			30 (30.93%)		
Military characteristics (Years Served)			13.39	8.30		15.00	10.31		12.94	8.12		13.98	8.23
ITQ-PTSD			15.88	5.77		18.00	3.45		18.65	3.80		9.79	4.75
ITQ-C-PTSD			32.35	10.84		29.29	8.64		38.27	6.69		20.98	8.52
ITQ-DSO			16.46	6.09		11.29	7.23		19.61	3.74		11.19	5.26
YBOCS			16.91	8.49		15.43	7.76		15.60	7.83		11.76	7.47

Note. *N* = 315. Due to missing data, not all frequencies total to 315. PTSD = Post-Traumatic Stress Disorder; C-PTSD = Complex Post-Traumatic Stress Disorder

### Comparing OCD severity between diagnostic groups

Firstly, Levene’s test (*p* =.848) and normality checks were conducted, confirming that assumptions were met. A one-way between subjects ANOVA examined how predicted Y-BOCS scores differed between the diagnostic groups (probable PTSD, probable C-PTSD, and No PTSD). Since there were no significant demographic differences between the diagnostic groups, covariates were not included in the ANOVA. The main effect found a significant difference in Y-BOCS scores between diagnostic groups (*F*(2,312) = 33.93, *p* <.001), indicating OCD severity to differ between the three diagnostic groups. Continuing exploration of hypothesis 1, two independent linear regression models were conducted. Model one explored whether Y-BOCS scores were predicted by ITQ-PTSD scores in participants with probable PTSD and model two explored whether Y-BOCS scores were predicted by ITQ-C-PTSD scores in participants with probable C-PTSD. Model one revealed a non-significant association between ITQ-PTSD and Y-BOCS scores among veterans with probable PTSD (*b* = 0.50, *p* =.337, 95% CI: -0.49 to 1.48). Model two revealed a significant association in the probable C-PTSD group, where Y-BOCS scores were predicted to increase by 0.57 for every one unit increase in ITQ-C-PTSD scores (*b* = 0.57, *p* <.001, 95% CI: 0.42 to 0.71). See Table 2  for linear regression models These results significantly indicate as C-PTSD symptom severity increases, so does OCD severity. However, a similar pattern was not revealed for those in the probable PTSD group. This disparity highlights the nuanced relationship between C-PTSD, PTSD, and OCD severity within the context of military veterans.

**Table 2:** Linear regression models predicting Y-BOCS scores based on ITQ scores

Hypothesis	Linear Regression Model	Diagnosis Group	Predictor	Outcome	b	t	*p* -value	*r*-squared	95% CI	
									LL	UL
1	Simple	Probable PTSD	ITQ- PTSD Scores	Y-BOCS Scores	0.50	0.99	0.337	-0.00	-0.56	1.48
	Simple	Probable C-PTSD	ITQ- C-PTSD Scores	Y-BOCS Scores	0.57	7.75	< 0.001	0.23	0.42	0.71
2	Simple	Probable C-PTSD	ITQ PTSD scores	Y-BOCS Scores	0.76	5.54	< 0.001	0.13	0.49	1.03
	Simple	Probable C-PTSD	ITQ DSO scores	Y-BOCS Scores	1.03	7.91	< 0.001	0.20	0.77	1.29
	Multiple	Probable C-PTSD	ITQ PTSD scores	Y-BOCS Scores	0.27	1.70	0.09	0.25	-0.04	0.57
		Probable C-PTSD	ITQ DSO scores	Y-BOCS Scores	0.88	5.54	< 0.001		0.56	1.19

Note. ITQ = International Trauma Questionnaire; DSO = Disturbances of Self-Organisation. *b* represents standardised regression coefficients. r-squared represents adjusted r-squared. LL and UL represent lower and upper limits of a confidence interval respectively

### Associations between OCD Severity and C-PTSD’s clusters of symptoms

Two simple linear regression models assessed whether OCD severity was associated with PTSD and DSO symptoms for veterans with probable C-PTSD. The first model assessing whether ITQ-PTSD scores predicted Y-BOCS scored resulted in a significant positive relationship, with higher ITQ-PTSD scores associated with higher Y-BOCS scores (*b* = 0.76, *p* <.001, 95% CI: 0.49 to 1.03). The adjusted R^2^ was 0.13, indicating that 13% of the variance found in OCD severity was explained by PTSD symptoms. The second model exploring the relationship between ITQ-DSO and Y-BOCS, showed that for every one-unit increase in ITQ-DSO scores, Y-BOCS scores significantly increased by 1.03, (*b* = 1.03, *p* <.001, 95% CI: 0.77 to 1.29). An adjusted R^2^ suggested that 20% of the variance found in OCD severity is explained by DSO scores (see Table 2). Both models suggest that as the severity of PTSD and DSO symptoms increase, so does OCD severity.

Prior to conducting the multiple linear regression, assumption tests were conducted. Although a Pearson correlation analysis showed a moderate association between the predictors (*r* =.57), the Variance Inflation Factor values were 1.49 for both predictors. As such, there were no multicollinearity concerns. To enhance prediction accuracy, a multiple linear regression assessed OCD severity based on ITQ-PTSD and ITQ-DSO scores for veterans with probable C-PTSD. A significant multiple linear regression model found as DSO symptoms increased, predicted Y-BOCS scores also increased (*b* = 0.88, *p* <.001, 95% CI: 0.56 to 1.19). However, the model revealed the previously significant relationship between ITQ-PTSD scores and Y-BOCS was no longer significant (*b* = 0.27, *p* =.091, 95% CI: -0.04 to 0.57), see Table 2. This illustrates how PTSD symptoms alone do not significantly predict OCD severity once DSO symptoms are accounted for. An R^2^ of 0.242, suggests 24.2% of the variance found in Y-BOCS scores was explained by PTSD and DSO symptoms. Overall, the key finding indicates that in veterans with probable C-PTSD, DSO symptoms are a significant predictor of OCD severity, while PTSD symptoms alone do not significantly predict OCD severity when DSO symptoms are included in the model.

## Discussion

The main aim of this study was to explore the potential difference in OCD severity between treatment seeking UK military veterans with probable PTSD and probable C-PTSD. The ANOVA indicated a significant difference in OCD severity between the three diagnostic groups. Further analysis demonstrated OCD severity to significantly increase as C-PTSD symptom severity increased for veterans meeting probable C-PTSD diagnosis. While no significant association was found between OCD severity and PTSD scores in the probable PTSD group, the absence of such a relationship cannot be confidently concluded due to the smaller sample size. The substantial sample size difference between the two diagnostic groups may offer insight into why there was no significant association in the probable PTSD group. Nonetheless, given the relatively limited research exploring OCD severity within military veterans, this study adds insight into how OCD severity may differ in veterans with probable PTSD and probable C-PTSD and may inform future research.

To our knowledge, no research has previously explored the difference in OCD severity between individuals with PTSD and C-PTSD. Thus, the current study provides novel findings. Before the differentiation between PTSD and C-PTSD as outlined in the ICD-11, individuals presenting with both conditions would have been classified under the diagnosis of PTSD. Therefore, to an extent, the findings support prior literature presenting the significant relationship between PTSD and OCD in the general and military population [42, 43, 44]. Furthermore, they support the premise of C-PTSD being related to greater functional impairment compared to PTSD and indicate potential disparities between the conditions [45]. This highlights the importance of considering comorbid OCD when providing treatment options for C-PTSD.

This current study found that, contrary to initial predictions, only DSO symptoms were significantly associated with OCD severity in C-PTSD, while PTSD symptoms alone were not. The multiple regression model demonstrated the potential collinearity between PTSD and DSO symptoms. It was outside the scope of this study to explain precisely how PTSD and DSO symptoms relate to one another to predict OCD; however, the DSO symptoms appear to have an important relationship with OCD severity within military veterans. Due to the symptoms within the DSO cluster, this evidence extends previous literature demonstrating how negative self-concept and interpersonal challenges likely influence OCD severity [30, 31, 33]. In light of the cognitive theory of obsessions, self-focused symptoms in the DSO cluster may prompt individuals to interpret intrusive thought’s as personally meaningful, potentially leading to OCD symptomology [9, 10]. This may provide insight into the association between DSO symptoms and OCD severity.

A possible explanation of the main findings, suggest the difference in OCD severity between probable PTSD and probable C-PTSD may occur due to veterans with probable C-PTSD presenting with greater functional impairment [17]. This hypothesis is supported by previous evidence signifying both C-PTSD and OCD as being related to functional impairment, with C-PTSD being related to greater functional impairment compared to PTSD [46, 47, 48]. Nonetheless, it may seem plausible that the functional impairment within both C-PTSD and OCD may exacerbate symptoms, creating a cyclical pattern of impairment. This premise aligns with research indicating how comorbid OCD is associated with greater functional impairment and lower quality of life compared to individuals only with OCD [33, 49, 50]. More still, some research indicates multiple combat deployments to be associated to greater levels of functional impairment, underpinning potential vulnerabilities towards psychiatric conditions within the military population [51].

A long-established relationship between trauma, PTSD and OCD has been recognised, with evidence indicating that the development of OCD may be trauma-related within veterans [44]. Some previous research proposes a post-traumatic subtype of OCD, due to evidence indicating OCD related to PTSD presenting distinct clinical features [52]. One example of trauma-related OCD shows that negative appraisals after a traumatic event can increase a person’s sense of responsibility to prevent harm, potentially triggering OCD [54]. Additionally, a meta-analysis has illustrated how multiple types of past interpersonal trauma including violence, neglect, emotional and sexual abuse, were associated with greater obsessive-compulsive symptom severity [30]. These types of traumas are more commonly associated with C-PTSD than PTSD [53, 54].

Additionally, the experience of moral injury has been associated more with C-PTSD than PTSD, with moral injury having a larger impact on DSO symptoms compared to PTSD symptoms, demonstrating the complex interplay between types of traumas and C-PTSD’s cluster of symptoms [24, 55]. This is particularly important within military veterans due to their line of work, whereby the prevalence of moral injury is greater than the general population [56]. This premise could help underpin the higher rates of C-PTSD compared to PTSD within this current study. It seems plausible to suggest the types of traumas experienced by military veterans could partly explain why the current study found OCD severity to be higher within the C-PTSD group compared to PTSD, as more complex traumas are associated with both C-PTSD and greater OCD severity. It would be beneficial for further research to explore how different types of traumas within veterans with C-PTSD relate to OCD acuteness.

Some previous literature proposes that the associations found between PTSD and OCD are due to overlapping symptomologies [42]. For example, defining features of OCD; unwanted thoughts and images, repetitive behaviours and avoidance of triggers are also found in PTSD [57]. The desire to supress unwanted thoughts is also found in OCD, PTSD and depression, further demonstrating the symptom overlap between psychiatric conditions [58]. However, this raises questions as to why only C-PTSD was associated with OCD and not PTSD in the current study, despite their apparent symptom overlap. One possible explanation of this difference may be attributed to the small sample in the probable PTSD, potentially limiting the statistical power to detect an association. Nonetheless, a valuable approach exploring overlapping symptoms is through network analysis, which can unravel so-called “bridge symptoms” hypothesised to explain comorbidity [59]. For example, network analysis has revealed how central symptoms of OCD, including distorted beliefs about one’s thoughts predicted depression and anxiety, with obsessions in OCD and guilt in depression acting as bridging symptoms between the conditions [60, 61]. Consequently, it is plausible the associations found within this current study could be partly explained by bridging symptoms found between these psychiatric conditions. Network analysis exploring C-PTSD and OCD would provide scope for future research.

This study’s findings contribute to the growth of research informing clinical treatment options for treatment seeking military veterans. The findings demonstrate the importance of distinguishing between PTSD and C-PTSD in clinical assessment and treatment planning. Clinicians should be aware that military veterans with probable C-PTSD may experience greater OCD severity compared to those with probable PTSD. However, the specific association in the probable PTSD group is unclear given the small sample size. Importantly, evidence indicates OCD may often be unrecognised and inadequately treated within veterans [3]. Considering prior literature and the current study’s findings depicting how as the severity of one psychiatric condition increases, so does the other, it emphasises the importance of recognising both conditions when evaluating treatment options for military veterans.

The exploration of comorbidities within military veterans when discussing treatment options is particularly important in relation to the DSO cluster of symptoms within C-PTSD, as these were more strongly associated to OCD severity. Addressing DSO symptoms in treatment planning could be imperative for positive treatment outcomes. For example, DSO symptoms are associated with interpersonal challenges, highlighting the importance of a strong therapeutic alliance [54]. A flexible modular approach to treating C-PTSD proposes how treatment should be broken down into different modules, targeting patient’s specific symptoms [62]. Due to C-PTSD’s distinct symptom clusters, this personalised approach encourages both the patient and clinician to choose suitable interventions focusing on the most problematic symptom clusters. In the context of the current study, the seemingly complex relationship between OCD and probable C-PTSD suggests a modular approach may best support patient outcomes, as it can focus on flexibility and targeted interventions based around the most challenging symptoms. This is particularly important in military veterans due to the high prevalence of trauma-related disorders and complex clinical presentations seemingly due to prolonged exposure to interpersonal experiences, such as war [63]. The combination of these findings presses the importance of exploring comorbid C-PTSD for military veterans seeking treatment for OCD, whilst also suggesting a holistic and modular treatment approach may be most beneficial for patient outcomes, due to the often complex and unique clinical profiles of military veterans [64].

### Strengths and limitations

This study has generated important novel findings highlighting the significant differences in OCD severity in veterans with probable PTSD and probable C-PTSD. Though this study indicates the association between OCD symptoms and C-PTSD symptoms, the sample size disparity between the diagnostic groups means we cannot confidently conclude the absence of a relationship between OCD symptoms and PTSD symptoms. The results also reveal the apparently greater associations between DSO symptoms and OCD severity compared to PTSD symptoms and OCD, for military veterans with probable C-PTSD. To achieve this, it used established standard psychometric scales to assess mental health conditions, enhancing the validity of the psychiatric conditions explored.

There are also some limitations which must be considered. Importantly, there were only 22 participants with probable PTSD in the sample. The large disparity in sample size between the probable PTSD and probable C-PTSD groups may have resulted in insufficient power to detect an effect in the probable PTSD group. While the findings demonstrate a relationship between OCD symptoms and C-PTSD symptoms, the small sample in the probable PTSD group limits our ability to confidently conclude the absence of a relationship between OCD symptoms and probable PTSD. This is particularly relevant given that PTSD scores did predict OCD symptoms within the probable C-PTSD group.

Additionally, a significant limitation of the current study is the reliance on self-report measures to diagnose PTSD and C-PTSD, which may lead to invalid diagnostic rates compared to true prevalence estimates. Self-report measures are susceptible to biases such as recall bias and lack of clinical oversite. As such, the diagnoses provided should be considered probable rather than definitive. The absence of clinician-administered measures or interviews may have contributed to inflated or invalid groupings, as self-report measures do not provide the opportunity for clinical judgement. Additionally, some discrepancy between self-reported and clinician-administered versions of the Y-BOCS scale has been identified, with clinicians appearing to rate OCD more severe than self-reported measures [65, 66]; indicating the possibility of OCD severity being inaccurately reported within this study. Moreover, some evidence suggests self-reported measures should be used in caution when exploring PTSD and OCD due to individuals finding it potentially challenging to differentiate between symptoms [67]. The self-report scales used in the current study might inaccurately attribute symptoms of one condition to another.

Furthermore, due to this study’s cross-sectional design it is not possible to determine causality, thus only associations can be inferred. The methodology also limits exploration of whether veterans’ OCD symptoms preceded trauma exposure or PTSD, making it difficult to determine temporality. Additionally, the study may have encompassed non-response bias due to many non-responders of the survey. It is plausible the non-responders presented with worse or better mental health resulting in findings underestimating or overestimating the relationship between probable C-PTSD and OCD. Similarly, given the focus on a clinical population of treatment-seeking veterans, the findings may not be generalisable to the broader veteran community or other clinical groups with PTSD and C-PTSD. Veterans actively seeking treatment may represent a distinct subset, potentially experiencing more severe symptoms that prompt them to seek help, compared to those in the wider community who may have less severe or different symptom profiles. Moreover, the study comprised of mainly males, making it difficult to generalise findings across sex. This is particularly important as evidence indicates females in U.K. Armed Forces are at less risk of experiencing mental health challenges [68]. In comparison, a meta-analysis revealed being female was a risk factor towards combat-related PTSD [69]. The limitations of this study therefore restrict the extent to which the findings can be generalised.

### Future directions

Due to the novel but limiting findings of this study, future research should focus on replication with larger samples on veteran and general populations, comprising more diverse population characteristics including sex and ethnicity. This will yield more reliable and generalisable findings. Future research should incorporate clinician administered measures in addition to self-report measures to enhance the accuracy of diagnostic groupings. Additionally, exploring the potential symptom overlap between OCD and C-PTSD, it would be beneficial to conduct network analysis to explore central and co-occurring symptoms between the conditions. This could guide treatment options by identifying bridging symptoms as potential targets for innovative interventions [70].

Exploring whether different types of traumas influence the association between C-PTSD and OCD could help inform preventative and treatment options. For example, investigating how different types of military trauma experienced during service influence the association between C-PTSD and OCD, could help identify veterans at risk of developing these psychiatric conditions. Also, OCD has distinct symptom dimensions, including contamination, ordering, hoarding, intrusive thoughts, forbidden thoughts and harm-related obsessions [71]. Therefore, it would be beneficial to explore whether C-PTSD relates to specific OCD domains. This could inform specialised treatment within effected symptom dimensions, whilst providing psychoeducation helping individuals understand the interplay between their symptoms. Lastly, to infer temporality, longitudinal research could be conducted to identify whether C-PTSD leads to OCD, or whether perhaps the symptoms from both conditions represent a continuum of functional impairment and psychological distress.

## Conclusion

In conclusion, treatment seeking UK military veterans with probable C-PTSD experienced greater OCD severity when C-PTSD symptoms increased. Though no association was identified in the probable PTSD group, the small sample means we cannot confidently conclude there was no association between OCD severity and symptoms of PTSD. For veterans with probable C-PTSD, DSO symptoms were more strongly associated with OCD severity compared to PTSD symptoms; further exemplifying the complex interplay between C-PTSD symptoms and OCD. Though currently the lack of replication and limitations of this study prevent drawing conclusions, they highlight interesting results that should be further explored. Future research should focus on replicating findings in a larger population of military veteran while incorporating clinician-administered assessments and explore how different types of traumas may influence OCD severity and how C-PTSD influences different OCD symptom dimensions.

## Data Availability

Data availability statement: Data are available upon reasonable request.
